# Supplementary material for: Impact of Induction Immunosuppressants on T Lymphocyte Subsets after Kidney Transplantation: A Prospective Observational Study with Focus on Anti-Thymocyte Globulin and Basiliximab Induction Therapies
Source: Int J Mol Sci. 2023 Sep 19;24(18):14288. doi: 10.3390/ijms241814288 (PMC10532255; doi:10.3390/ijms241814288)
Supplement: Supplementary file 1 [file ijms-24-14288-s001.zip › ijms-2559876-supplementary.pdf]

Supplementary Tables and Figures

Table S1. Baseline characteristics of validation cohort

	**ATG (*n* = 5)**	**BXM (*n* = 8)**
Age (years)	46.6±7.8	39.5±9.5
Male, *n* (%)	3 (60.0)	5 (62.5)
Donor age (years)	35.8±13.8	53.7±11.3
Donor type (LD), *n* (%)	1 (20.0)	5 (62.5)
HLA mismatch number	4.4±0.8	3.0±1.6
PRA positive, *n* (%)	2 (40.0)	2 (25.0)
PRA Class I	16.6±22.0	13.5±29.5
PRA Class II	0±0	0±0
DS-HLA Antibody, *n* (%)	2 (40.0)	1 (12.5)
Rituximab Use, *n* (%)	0 (0)	2 (25.0)

Data are expressed as means ± standard deviations or number (percentage). Abbreviations: ATG, anti-thymocyte globulin; DS-HLA, donor-specific human leukocyte antigen; BXM, basiliximab.

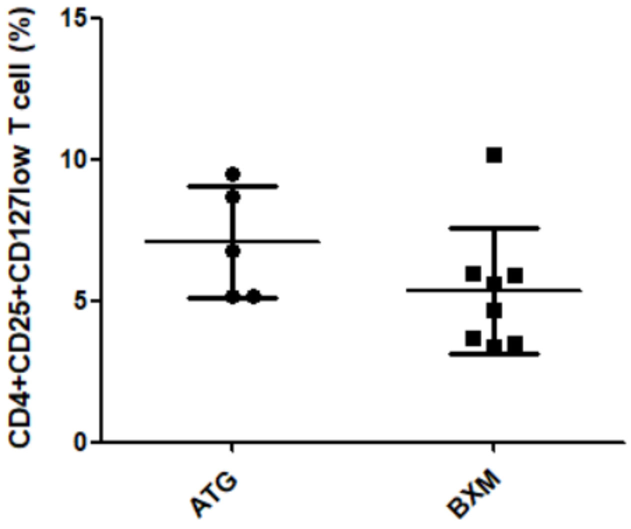

Figure S1. Comparison of CD4+CD25+CD127low T cell expression in validation cohort.

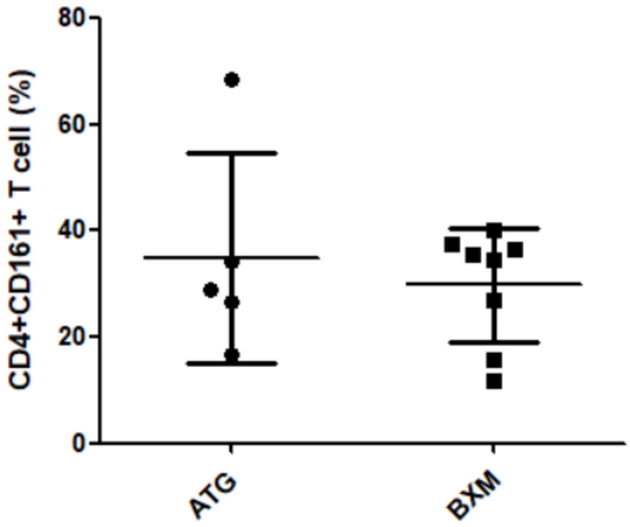

Figure S2. Comparison of CD4+CD161+ T cell expression in validation cohort.
